# Correction: ERdj8 governs the size of autophagosomes during the formation process

**DOI:** 10.1083/jcb.20190312707282021c

**Published:** 2021-08-06

**Authors:** Yo-hei Yamamoto, Ayano Kasai, Hiroko Omori, Tomoe Takino, Munechika Sugihara, Tetsuo Umemoto, Maho Hamasaki, Tomohisa Hatta, Tohru Natsume, Richard I. Morimoto, Ritsuko Arai, Satoshi Waguri, Miyuki Sato, Ken Sato, Shoshana Bar-Nun, Tamotsu Yoshimori, Takeshi Noda, Kazuhiro Nagata

Vol. 219, No. 8 | 10.1083/jcb.201903127 | June 3, 2020

After publication, the authors noticed an error in Fig. 5, A–C. In the experiments depicted in these panels, the authors used an antibody against succinate dehydrogenase complex, subunit A (SDHA)—also known as Complex II—as a marker of the mitochondrial inner membrane, not COX-II (cytochrome c oxidase, subunit II), as originally reported in the paper. The corrected figure panels are shown here.

**Figure:**
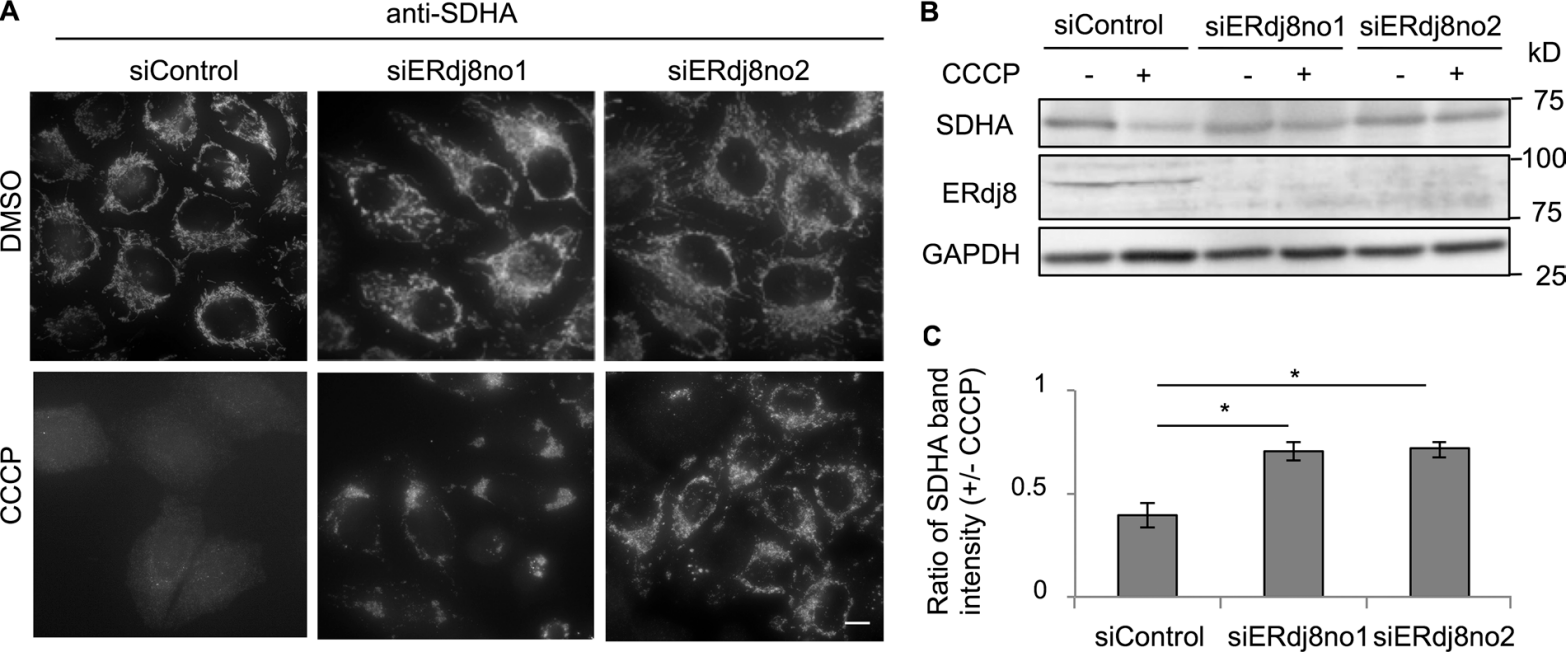


The figure legend, Results, and Materials and methods have also been corrected. Changes are indicated with underlined text as follows:

Figure 5. **ERdj8 depletion causes a defect in the enwrapping of larger mitochondria. (A–C)** HeLa cells expressing mCherry-Parkin were treated with siRNA (siERdj8no1, siERdj8no2, and control). The cells were treated with CCCP or DMSO for 20 h, immunostained with SDHA antibody, and imaged on a DeltaVision system. Scale bar, 10 μm. **(B)** The lysates were subjected to immunoblotting. **(C)** The average and SEM of three independent experiments of the ratio of SDHA to GAPDH band intensity. *, P < 0.05.

## Results

### ERdj8 depletion allows enwrapping of small paternal mitochondria, but not normal somatic mitochondria

CCCP treatment attenuated the signal from the mitochondria inner membrane protein succinate dehydrogenase A (SDHA), but this attenuation was suppressed in ERdj8-knockdown cells (Fig. 5, A–C).

## Materials and methods

### Antibodies

The following primary antibodies were obtained from the indicated suppliers: rabbit anti-DNAJC16 (ERdj8; Proteintech, 17599-1-AP; Western blotting [WB], 1/500); mouse anti-ATG13 (Merck, MABC46; immunocytochemistry [IC], 1/100), mouse anti-SDHA (Novex, 459200; WB, IC, 1/1,000).

### Mitophagy assay

After 48 h, the cells were treated with 5 μM CCCP (Sigma, C2759-250MG) for 20 h to induce mitophagy.

These changes do not alter the interpretation of the figure or the conclusions of the manuscript. The errors appear only in print and in PDF versions downloaded on or before August 2, 2021.

